# Magnetic Resonance Imaging and Computed Tomography for the Noninvasive Assessment of Arterial Aging: A Review by the VascAgeNet COST Action

**DOI:** 10.1161/JAHA.122.027414

**Published:** 2023-05-15

**Authors:** Elisabetta Bianchini, Mai Tone Lønnebakken, Peter Wohlfahrt, Senol Piskin, Dimitrios Terentes‐Printzios, Jordi Alastruey, Andrea Guala

**Affiliations:** ^1^ Institute of Clinical Physiology CNR Pisa Italy; ^2^ Department of Clinical Science University of Bergen Bergen Norway; ^3^ Department of Heart Disease Haukeland University Hospital Bergen Norway; ^4^ Department of Preventive Cardiology Institute for Clinical and Experimental Medicine Prague Czech Republic; ^5^ Centre for Cardiovascular Prevention Charles University Medical School I and Thomayer Hospital Prague Czech Republic; ^6^ Department of Medicine II Charles University in Prague, First Faculty of Medicine Prague Czech Republic; ^7^ Department of Mechanical Engineering, Faculty of Engineering and Natural Sciences Istinye University Istanbul Turkey; ^8^ Modeling, Simulation and Extended Reality Laboratory Istinye University Istanbul Turkey; ^9^ First Department of Cardiology, Hippokration Hospital, Athens Medical School National and Kapodistrian University of Athens Greece; ^10^ School of Biomedical Engineering and Imaging Sciences King’s College London London UK; ^11^ Vall d’Hebron Institut de Recerca (VHIR) Barcelona Spain; ^12^ CIBER‐CV, Instituto de Salud Carlos III Madrid Spain

**Keywords:** aging, aorta, arterial stiffness, arteries, calcification, Cardiovascular Disease, Computerized Tomography (CT), Magnetic Resonance Imaging (MRI), Nuclear Cardiology and PET, Imaging

## Abstract

Magnetic resonance imaging and computed tomography allow the characterization of arterial state and function with high confidence and thus play a key role in the understanding of arterial aging and its translation into the clinic. Decades of research into the development of innovative imaging sequences and image analysis techniques have led to the identification of a large number of potential biomarkers, some bringing improvement in basic science, others in clinical practice. Nonetheless, the complexity of some of these biomarkers and the image analysis techniques required for their computation hamper their widespread use. In this narrative review, current biomarkers related to aging of the aorta, their founding principles, the sequence, and postprocessing required, and their predictive values for cardiovascular events are summarized. For each biomarker a summary of reference values and reproducibility studies and limitations is provided. The present review, developed in the COST Action VascAgeNet, aims to guide clinicians and technical researchers in the critical understanding of the possibilities offered by these advanced imaging modalities for studying the state and function of the aorta, and their possible clinically relevant relationships with aging.

**Nonstandard Abbreviations and Acronyms**
HU: Hounsfield units
PC: phase contrast
PWV: pulse wave velocity

Magnetic resonance imaging (MRI) and computed tomography (CT) are advanced imaging modalities providing a wide range of possibilities to study the cardiovascular system. Despite differing in many ways, both techniques allow for excellent visualization of deep arteries, thus providing a window over their structure and function. This is particularly relevant for the study of arterial aging as arterial dimensions, shape, and mechanical properties as well as the increasing presence of calcium plaques are among the most described arterial characteristics associated with age.

The present narrative review aims to firstly introduce the reader to the basics of the functioning of MRI and CT, providing the interested reader with further specialized literature. Then, in‐depth descriptions of the most important quantities related to aortic aging are provided. For each potential biomarker, a brief introduction on the physical or clinical principle supporting its meaning as a descriptor of arterial state is followed by a summary of its predictive value for relevant end points. Key information on the required image characteristics and their analysis, as well as their association with age, is briefly described. Lastly, when available, reference values and reproducibility studies are reported along with a list of current limitations.

## MRI AND CT FUNCTIONING

### Magnetic Resonance Imaging

MRI measures the magnetic properties of tissues and its first medical application was realized in 1973. Most medical applications are focused on visualizing hydrogen nuclei, which form the majority of atoms in the body and contains a single proton that rotates (spinning). The rotation of the unpaired proton creates a magnetic moment, which is exploited to create images. The magnetic moment orientation is normally casual, but when an external static magnetic field is applied, the magnetic moment orients in a number of directions that are related to the spin number. Moreover, because of the spinning, the nuclei will “rotate” around the axis of the magnetic field originating a precession movement, whose frequency (called Larmor) is proportional to the applied field. In MRI, precession is usually created by a strong magnetic field, conventionally in the *Z* axis, along the long axis of the patient. Most of nuclei will precess aligned with it (the low energy state) creating a net longitudinal magnetization in the *Z*‐axis direction. A further magnetic field is obtained by means of radiofrequency waves, creating an oscillating transversal component of the magnetization. When radiofrequency are switched off the nuclei relax back to their resting state (relaxation). The magnetic field changes associated with relaxation induce electric current in the receiving coils, providing information about proton density in specific biological tissues and thus forming the basis of the visualization of different structures. Two properties are measured: spin–lattice relaxation (T1 recovery), the return of the longitudinal component of the net magnetization vector to its original value, and spin–spin relaxation (T2 decay), the disappearance of the transverse component of the net magnetization vector. Data are collected in the Fourier (or k‐) space, whose inverse transformation provides the reconstructed image. The brightness of the pixel is the amplitude of the returned signal, which represents the weighted proton density distribution, and depends on the tissue‐related parameters T1 and T2.

Besides imaging structures, MRI provides blood velocity data via phase‐contrast (PC) imaging: this approach exploits information about the changes in angle of moving protons that result in changes in the phase of the received signal. Two equal and opposite gradients are applied: stationary protons will not undergo phase shift, whereas mobile protons will show phase shift because their position with respect to the transmitted signal is changing. The degree of phase shift is directly related with the velocity of the mobile protons. Hence, in the reconstruction of the received signal, only protons that are moving will contribute with a phase shift and these local phase shift values can be used to assess local velocity. PC imaging is referred to as “in‐plane” when the measured velocities are directed as the image plane main axes or “through‐plane” when the velocity measured is perpendicular to the image plane.

The spatial and temporal resolutions of MRI vary substantially depending on clinical needs, sequences and their setting, and limits on the duration of the acquisition and on patients' acceptance and capacity for breath‐holding. Overall, most of MRI images have a spatial resolution in the order of 1 to 3 mm, while the temporal resolution is typically between 20 and 40 ms.

MRI is considered safe since it is based on nonionizing radiofrequency waves; the absorbed radiofrequency energy increases the vibrations of tissue elements, and results in small temperature increases. Another aspect to be mentioned is the incompatibly of the technique with ferromagnetic objects. Advances in MRI technology useful for clinical applications are focused on image quality and shorter acquisition times. This can be obtained with further pulse sequences, higher gradients, and external magnetic fields, new coils geometry, and reconstruction algorithms.

The main limitation of MRI is related to its high cost, which arises from the scanner itself and the needed infrastructure and skilled technicians, resulting in limited availability. Moreover, virtually all MRI scanners are not portable. Finally, patient discomfort might occur, especially because of claustrophobia and difficulty of holding breath, while in some cases there is a need for the use of external contrast agents (materials with high magnetic susceptibility, modifying T1 and T2), whose risk of side effects should be considered. Irregular heartbeats and tachycardia also represent a problem for ECG‐gated acquisitions.

### Computed Tomography

CT provides images of the x‐ray attenuation properties of the tissues and its first medical application was realized in the 1970s. X‐rays are produced and directed by an x‐ray tube, are attenuated by the patient's tissues and measured by a detector. Attenuation coefficient represents the probability of interaction between a photon and matter and differs in different tissues, this allows for the recognition of different structures. To obtain images, x‐ray beams are sent through the body as single lines. The acquisition is repeated for a large number of angles and distances from the imaged part so that attenuation measurements for many points of an analyzed slice are obtained, covering the entire field of view. Depending on how x‐ray beams are generated and detected, different types of scanners are available. In a*xial transverse tomography* (2‐dimensional [2D]), the film is positioned horizontally in front of the patient and slightly below the focal plane. Both the patient and the detector rotate at the same fixed speed around a vertical axis while the x‐ray source remains stationary. More recently, *helical and multislice CT* have been introduced, allowing for 3‐dimensional (3D) and 4‐dimensional (4D) imaging. For helical systems the x‐ray tube rotates continuously around the patient while the patient is slowly translated through the gantry, hence describing a helical orbit with respect to the patient. In multislice CT the detector array consists of multiple detector rows, measuring several slices per rotation of the x‐ray tube. CT scanners provide digital images that are based on single elements called pixels, each expressed in Hounsfield units (HU), a linear transformation of attenuation values in which 0 HU represent the radiodensity of distilled water. Contrast agents (or dyes) can be used in CT imaging when attenuation differences between tissues are too small to distinguish. They are substances with a high attenuation coefficient and can be used for intravascular (blood vessels, heart cavities) and intracavitary (kidney, bladder, etc.) applications. The spatial resolution of CT images is generally high, on the order of 0.5 to 2 mm, and depends on several factors. On the other hand, because of the intrinsic function, the *temporal resolution* is a critical point, being generally limited.

The main limitation of CT is that it is based on ionizing radiation and radiation doses are relatively high (for example a whole‐body screening has an approximate dose of 7 mSv, comparable with the natural exposure of around 4–9 months on earth). Despite a longstanding lowering trend in radiation exposure attributable to technological improvements in the last decades, the exposure to radiation still represents a limiting factor, especially for serial assessment, and a trade‐off between good images quality and low radiation dose has to be found. Moreover, contrast agents are frequently adopted, and the consequent risk of side effects should be considered. Irregular heartbeats and tachycardia also represent a problem for ECG‐gated CT acquisition.

#### Further Reading

Further details on the functioning of MRI and CT are available in the following books:

- Suetens, P (2009). *Fundamentals of Medical Imaging* (2nd ed.). (Cambridge University Press).
- Barrie Smith, N and Webb, A (2010). *Introduction to Medical Imaging: Physics, Engineering and Clinical Applications*. (Cambridge University Press).
- Bushberg, JT; Seibert, JA; Leidholdt, EM; Boone, JM (2020). *The Essential Physics of Medical Imaging* (4th ed.). (Lippincott Williams & Wilkins).
- Dale, BM; Brown, MA; Semelka, RC (2015). *MRI: Basic Principles and Applications*. (Wiley‐Blackwell).
- Mamourian, AC (2013). *CT Imaging: Practical Physics, Artifacts, and Pitfalls*. (Oxford University Press).
- Westbrook, C and Talbot, J (2018). *MRI in Practice* (5th Edition). (Wiley‐Blackwell)

**Figure 1:**
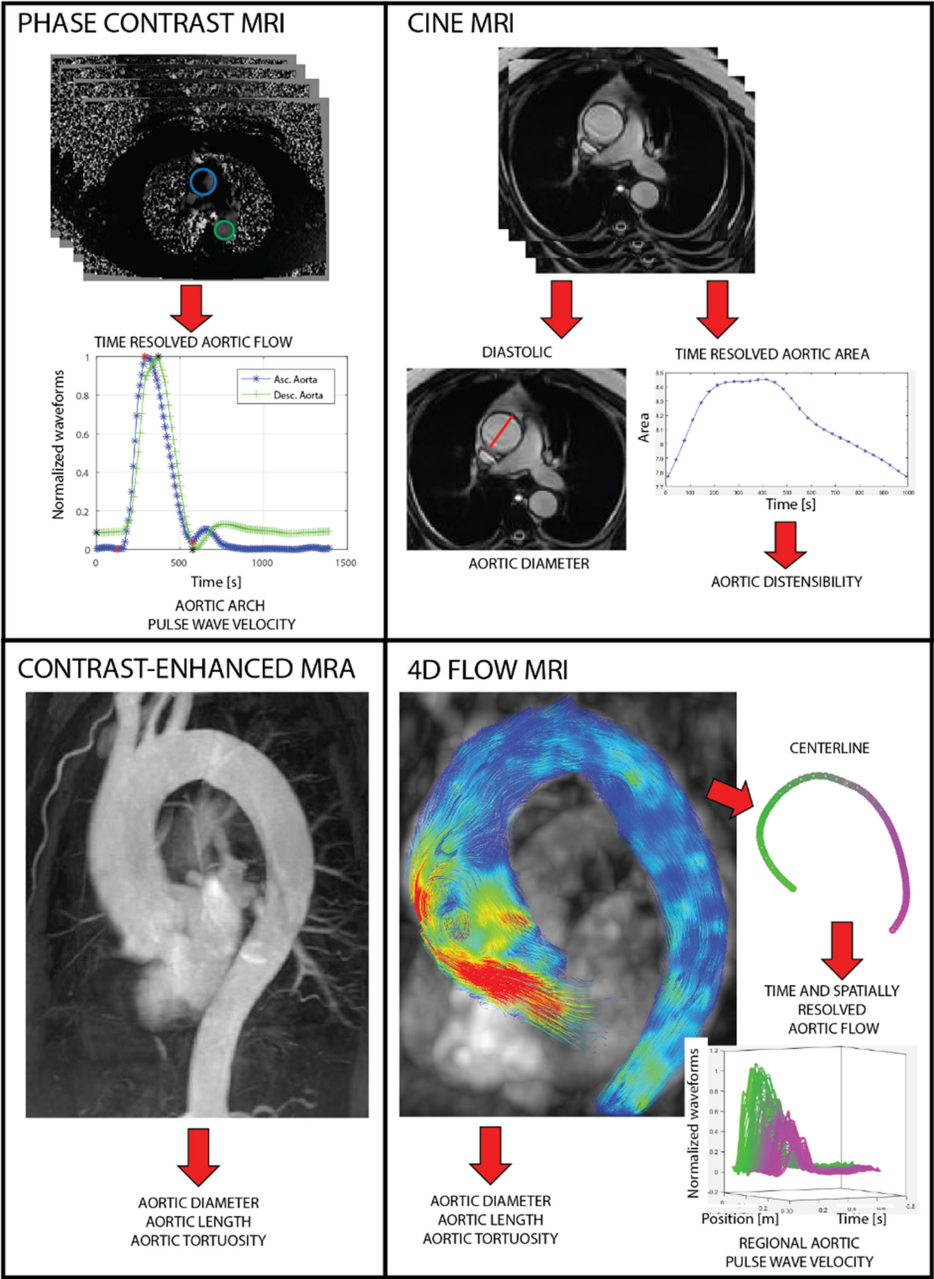
Phase‐contrast magnetic resonance imaging with 1 velocity encoding (top left, useful to compute aortic arch pulse wave velocity), cine magnetic resonance imaging (top right, used to assess aortic diameter, distensibility and circumferential strain), contrast‐enhanced magnetic resonance angiography (bottom left, useful for the quantification of aortic size and shape), and 4‐dimensional flow magnetic resonance imaging (bottom right, used to quantify regional pulse wave velocity). 4D indicates 4‐dimensional; MRA, magnetic resonance angiography; and MRI, magnetic resonance imaging.

**Figure 2:**
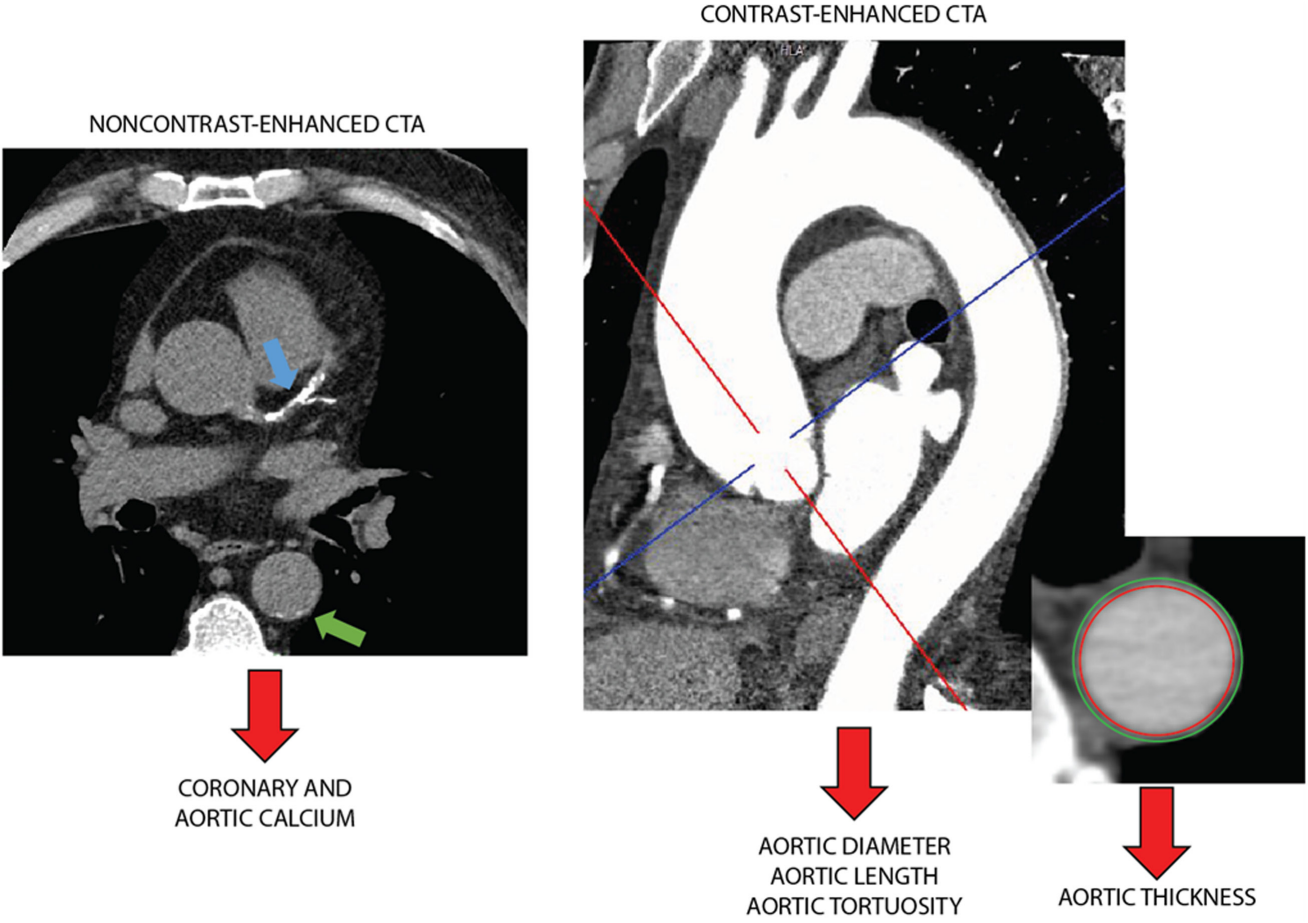
From left to right: coronary angiogram (showing coronary artery [blue arrow] and aortic [green arrow] calcium), contrast‐enhanced computed tomography angiography (useful for the quantification of aortic size and shape), and a multiplanar reformation (useful for aortic wall assessment). CTA indicates computed tomography angiography.

An overview of images collected using MRI and CT is provided in Figures 1 and 2, respectively.

## BIOMARKERS OF AORTIC AGING

This section describes the most important quantities measurable by MRI or CT that are related to aging of the aorta.^1^ For each potential biomarker, a brief introduction is followed by key information about the required imaging sequences and image analysis, as well as by results from studies assessing the relationship with aging, reference values and reproducibility. We conclude the description of each quantity with a list of its limitations. For each potential biomarker, key information is summarized in the Table. Relevant descriptors of arterial aging measured in more peripheral arteries, such as the carotid and femoral arteries, are mainly studied by ultrasound, and thus fall beyond the scope of this review.

**Table 1:** Summary of Clinical Evidence, Imaging Technique and Analysis, Reproducibility, and Reference Values

	Predicts cardiovascular events	Imaging technique	Target	Sequence	Established image analysis	Reproducibility			Reference value	
						Intra‐obs.	Inter‐obs.	Scan‐rescan	Adults	Children
Pulse wave velocity, m/s	Yes	MRI	Stiffness	Phase‐contrast in 1 or 3 directions	No	Yes	Yes	Yes	Yes	Yes
Coronary calcium score	Yes	CT	Calcification	ECG‐gated noncontrast CT (specific setting)	Yes	Yes	Yes	Yes	Yes	No
Diameter, mm	Yes	CT, MRI	Size	Many 2D or 3D (see text)	Yes	Yes	Yes	Yes	Yes	Yes
Wall thickness, mm	Untested	CT, MRI	Size	Many 2D or 3D (see text)	No	Yes	Yes	Yes	No	No
Length, mm	Untested	CT, MRI	Size	Many 2D or 3D (see text)	Yes	Yes	Yes	Yes	No	No
Tortuosity	Untested	CT, MRI	Shape	Many 2D or 3D (see text)	Yes	Yes	Yes	Yes	No	No
Distensibility	Yes	CT, MRI	Stiffness	Many time‐resolved (see text)	Yes	Yes	Yes	Yes	Yes	Yes
Aortic strain, %	Untested	CT, MRI	Stiffness	Many time‐resolved (see text)	Yes (circumferential) No (longitudinal)	Yes (circumferential) Yes (longitudinal)	Yes (circumferential) Yes (longitudinal)	No (circumferential) No (longitudinal)	No	No

2D indicates 2‐dimensional; 3D, 3‐dimensional; CT, computed tomography; and MRI, magnetic resonance imaging.

### Pulse Wave Velocity

Pulse wave velocity (PWV) is the velocity of propagation of waves. As waves travel faster in a more rigid environment, PWV is widely considered to be positively related to wall stiffness. Direct assessment of PWV with imaging is performed by tracking blood velocity waveforms. Of note, given the Nyquist–Shannon sampling theorem, the temporal resolution of the velocity data put an upper limit on the capacity to quantify PWV, both in terms of length over which it is assessed as well as in the maximum value that can be quantified. Thus, PWV can be measured by MRI but not CT.

#### Sequences

Phase‐contrast (PC) sequences are the most‐used MRI sequences to quantify PWV (Figure 1, right). They may differ in terms of the number of velocity‐encoding directions (one‐dimensional [1D], 2D, or 3D). One‐dimensional velocity encoding data are obtained by cross‐sectional PC images of a single 2D slice, where the through‐plane velocity is quantified. Two‐dimensional velocity data are obtained in‐plane (like anterior–posterior and superior–inferior velocity encoding in sagittal view),^2^ while 3D velocity encoding (often referred to as 4D flow) allows for complete flow field description, covering all possible velocity directions. An alternative to PC sequences is provided by M‐mode Fourier velocity encoding, which allows for high temporal resolution, thus enabling the computation of PWV over shorter segments, but has the shortcoming of requiring the vessel to be relatively straight.^3^

#### Assessment

Two quantities are needed to assess PWV: pulse wave transit time and traveled distance. Early studies used to quantify transit time by comparing velocity waveforms obtained via 1D through‐plane PC at 2 locations, mainly ascending and descending aorta, frequently with 1 PC image including the 2 sections, extracting the so‐called “arch PWV.”^2^, ^4^, ^5^, ^6^ More recently, a continuous coupling of transit time and location has gained ground because of the availability of 2‐ or 3D‐velocity encodings over a substantial length of the aorta and demonstrated better relationship with gold‐standard catheter,^2^ possibly for being less sensitive to temporal resolution^7^ because of the large number of data points.

Several methods to compute transit time have been proposed, tested, and compared. In the early studies, 2 methods were used. The earliest being the identification of a fiducial point, generally the “foot” of the wave, ie, the beginning of flow upslope, as the time of crossing between the linear fit of most of the upslope and the late diastolic mean velocity. This method suffers more from the limited temporal resolution, which often represents a limit in the assessment of PWV, compared with frequency domain methods.^5^ Other technique for fiducial point detection makes use of first or second temporal derivatives of the waveform. Another method for transit time assessment compares 2 entire waveforms and identify the time shift that results in the best correlation between them^4^, ^8^ or their Fourier transforms.^7^, ^9^ More recently, methods comparing the systolic upslope via temporal shift,^4^, ^6^ group‐delay^9^ or Wavelet analysis^5^ have gained ground. These methods offer the advantage of avoiding diastolic flow variability (a characteristic that affects methods that compare the entire waveforms) while making the most of the systolic propagation (as opposed to a single point, as it is done in methods relying on a fiducial point).

Propagation distance can be measured by means of a stack of 2D images or by 3D angiograms, normally via the computation of aortic centerline without substantial impact on PWV values^4^, ^10^ (see below). Gating may play a role, since proximal aorta length in diastole is lower than in systole attributable to aortic root motion.^10^, ^11^

#### Association With Age and Reference Values

Several studies identified a progressive increase in MRI‐derived PWV with age.^4^, ^5^, ^6^, ^7^, ^12^, ^13^, ^14^, ^15^, ^16^, ^17^, ^18^ This was mainly obtained by 2D PC with through‐plane velocity encoding^4^, ^5^, ^6^, ^12^, ^13^, ^16^ but also regionally by 4D‐flow.^7^, ^12^, ^14^, ^15^, ^17^, ^19^, ^20^, ^21^ Regarding transit time estimation techniques, PWV via upslope analysis in frequency domain was reported to provide the best association with age,^5^, ^19^ followed by whole‐waveform techniques,^4^, ^5^, ^19^ and further by fiducial point techniques.^4^ The extent of aorta stiffening with aging differs markedly between individual segments^7^, ^12^, ^13^, ^22^ and could be partially the result of the tendency for blood pressure to rise with age. Age‐ and sex‐related normative values of thoracic aorta PWV for children,^23^ adolescents,^23^ and adults^14^, ^24^ are available.

#### Accuracy and Reproducibility

Accuracy of MRI‐derived PWV was reported by comparison with values invasively assessed by pressure catheters in humans and phantoms. Grotenhuis et al reported good agreement between invasive PWV and values obtained with 2D PC images with a temporal resolution of 6 to 10 ms and foot‐to‐foot transit time in the aortic arch and descending aorta.^25^ Westenberg et al obtained good correlations between invasive values and PWV obtained by both in‐plane and through‐plane PC imaging with temporal resolution of 9 and 10 ms, respectively, with the former outperforming the latter.^2^ Four‐dimensional flow‐derived PWV was highly correlated to Peterson elastic modulus in humans^26^ and in a human tissue‐mimicking phantom^27^ with temporal resolutions of 25 and 20 ms, respectively. Marked correlation was also reported between PWV obtained by Fourier‐velocity encoding at a temporal resolution of 3.5 ms and invasive data in the descending aorta^22^ and in a tissue‐mimicking phantom.^3^

Many studies tested intra‐ and inter‐observer reproducibility and mostly reported good‐to‐excellent results. Reproducibility tests are available for Fourier‐encoded data,^3^, ^22^ 2D PC with both in‐ and through‐plane velocity encoding^2^, ^4^, ^8^, ^9^, ^13^, ^25^, ^28^ and for 4D flow.^14^, ^19^, ^20^, ^29^ Scan‐rescan (ie, test–retest, inter‐scan) reproducibility was good or moderate by 2D PC,^13^, ^28^ 4D flow^20^ and Fourier‐encoded data.^22^ Notably, the impact of the accuracy and reproducibility of traveling distance on the obtained PWV was reported to be limited.^10^

However, different sequences and strategies may not be comparable. Several studies showed similar PWV values comparing different techniques for transit time computation,^8^, ^15^, ^29^ with the exception of using peak velocity as fiducial point, which should be avoided.^15^ In a small study, comparing the whole waveform resulted in lower values compared with limiting the analysis to the sole upslope.^7^ Furthermore, 2D PC with in‐ versus through‐plane velocity encoding resulted in similar absolute values, but the latter was more reproducible^2^, ^8^ and in better agreement with invasive data.^2^ Several studies confirmed that a strategy where waveforms are extracted at many locations over a continuous domain may be preferred because of the (1) large number of extracted waveforms reduce the impact of noise^2^, ^7^, ^30^ and the (2) intimately shared absolute temporal reference given by a unique gating.^15^ Such a strategy was shown to be less influenced by temporal resolution.^19^ Similarly, techniques that exploit the upslope in the frequency domain may be less sensitive to temporal resolution compared with single fiducial point or time analysis of the whole upslope, resulting in improved reproducibility^5^, ^9^, ^19^ and correlation with age.^5^

#### Predictive Value

PWV measured over the aortic arch has been shown to be a significant predictor of cardiovascular events among middle‐aged individuals free from overt cardiovascular disease^31^ and of nonfatal extracardiac cardiovascular events in subjects without cardiovascular disease.^32^ Notably, these studies tested the prognostic value in multivariable models, including blood pressure.

#### Limitations

The limited availability of commercial software, possibly because of a lack of consensus on the best technique for transit time computation, and the time‐consuming postprocessing have limited the widespread use of PWV computed by MRI. Fully automatic solutions have been recently developed and may increase the use of PWV by MRI,^33^ while a growing number of commercial software include computer‐guided tools for PWV measure. Moreover, certain sequences, such as in‐plane 2D PC and 4D flow, still require substantial time to be acquired. This is particularly relevant as a compromise is needed between acquisition duration (and therefore cost and patient discomfort) and temporal resolution: a shorter acquisition duration results in lower temporal resolution, in turn precluding the assessment of PWV over short segments or in patients with particularly stiff arteries. Finally, more studies are needed to compare the predictive value of PWV obtained with different transit time assessment methods.

### Diameter, Thickness, Length, and Tortuosity

MRI and CT permit the visualization of deep vascular structures (Figures 1 and 2). As most of vasculature is composed by tubular segments, their main geometrical characteristics are diameter, wall thickness, length, and tortuosity. Tortuosity is a measure of vessel straightness computed as ratio of the centerline length to straight distance between ends. A larger value implies that the region of the aorta between inlet and outlet is less straight. The versatility of both MRI and CT and their high spatial resolution allow for the assessment of most large‐ and middle‐size vessels. However, the present review will primarily focus on the aorta.

#### Sequences

The sole, basic requirement for an MRI or CT sequence to be used for geometrical assessment is the delineation of the boundaries of the aorta. Consequently, a wide range of sequences are used. A key characteristic of the sequence is the number of spatial dimensions: 2D sequences allow for the assessment of a limited number of characteristics, such as diameter if they are acquired perpendicular to the vessel, and length if they cover the longitudinal section of a predominantly straight segment, but they are faster to acquire. Conversely, 3D sequences can be reformatted along any direction, providing virtually any perspective.

Regarding MRI, at least historically, 3D sequences required the administration of external contrast agent, such as gadolinium, which permits detailed 3D angiogram.^34^ As administration of gadolinium contrast is associated with certain risks, it is costly and acquisitions are often non‐gated, other sequences have been proposed. This is the case of 3D balanced steady‐state free precession, true fast imaging with steady‐state precession, and balanced fast‐field echo sequences, all offering high signal‐to‐noise ratio and good contrast. Other sequences, like time‐of‐flight and phase‐contrast enhanced angiography, rely on blood flow within the vessel to improve contrast. ECG and respiratory gating improve image quality, especially in region with substantial movement, such as the aortic root.^34^ For wall thickness assessment, classic spatial resolution of MRI is about half of the aortic wall thickness, being therefore susceptible to partial volume effects. However, it is worth noting that certain sequences, such as black‐blood spin echo T1‐ or T2‐weighted ECG gated acquisitions, provide sharp contrast between wall and blood and thus allow for wall thickness assessment.^35^, ^36^

Both contrast‐enhanced and noncontrast CT images provide excellent spatial resolution for geometric assessment of large and small arteries and can thus be used to assess diameter, length and tortuosity.^37^, ^38^ Conversely, contrast enhancement is required for wall thickness assessment.^39^, ^40^

#### Assessment

The assessment of aortic structure is most often obtained manually or semiautomatically. For all geometrical parameters, the identification of the location of measurement is fundamental. To this aim, anatomical landmarks should be used.^34^

Regarding diameter on 3D images, it should be measured at standardized levels determined by anatomical landmarks after double‐oblique multiplanar reformatting, taking care of obtaining an analysis plane perpendicular to artery main axis, possibly via the computation of vessel centerline.^34^ Both, inner (lumen) or outer (external vessel wall) diameter may be measured.^34^ Semiautomatic methods based on segmentation and 3D centerline analysis can be used.^41^ If 2D images are used, they need to be acquired perpendicular to the vessel.

As the aorta is not straight, centerline computation is fundamental for the assessment of length and tortuosity and should be obtained automatically after wall and inlet and outlet boundaries are defined, a task often requiring manual determination. Arterial length is then measured as the centerline distance between inlet and outlet boundaries points.^42^ Once intra‐arterial length is computed, its length can be divided by the straight distance between boundaries to obtain tortuosity. For aortic length assessment by MRI, a stack of 2D sagittal oblique planes as well as in 3D balanced steady‐state free precession or contract‐enhanced MRI angiography sequences can be used. Both contrast‐ and noncontrast‐enhanced CT images can be used to assess arterial length.

Contrast‐enhanced CT angiography images can be used to measure aortic wall thickness, either directly or measuring internal and external aortic diameters.^40^ In most of studies, aortic wall thickness by MRI is measured manually, with several measures along the circumference averaged,^17^, ^35^, ^36^ either directly^17^, ^35^, ^36^ or via the measurement of inner and outer diameter.^43^

#### Association With Age and Reference Values

Aortic diameter increases with age as reported by MRI^13^, ^44^ and CT^38^, ^45^, ^46^ and is larger in men compared with women.^45^, ^46^ The effect of aging on aorta dilatation is heterogeneous, being higher in the ascending compared with the abdominal aorta.^13^ Age, sex, and body surface area dependent reference values for aortic diameter determined by CT^46^, ^47^ as well as by MRI^48^ have been reported. Aorta length increases heterogeneously^16^, ^49^ with age as quantified by CT^38^, ^50^ and MRI.^13^, ^49^ The tortuosity of the descending aorta,^37^, ^51^ but not of the aortic arch,^52^ increases with aging. Contrasting results have been reported about eventual sex differences in descending aorta tortuosity.^37^, ^51^ Aortic wall thickness increases with age,^17^, ^35^, ^36^, ^40^, ^43^ and it is larger in men compared with women.^35^, ^36^, ^40^, ^43^ Both absolute wall thickness and the extent of its age‐dependence vary on the aortic segment analyzed.^40^, ^43^

#### Accuracy and Reproducibility

The reproducibility of aortic diameter measurements using many MRI sequences, such as 2D and 3D cardiac‐gated SSFP sequence, contrast‐enhanced magnetic resonance angiography and T2 black‐blood, is good to excellent.^53^, ^54^ Among MRI sequences, the 3D‐navigated, cardiac‐gated SSFP sequence was suggested as the most accurate and reproducible for diameters measurement.^53^ The reproducibility of aortic diameter measurements on contrast‐enhanced CT after multiplanar reconstruction is excellent.^41^, ^54^, ^55^ Similar performances were obtained with semiautomatic techniques based on the generation of centerline,^55^ while ECG gating, by eliminating motion artifacts and variability in diameter changes related to heart cycle, improves measurements.

Aortic length assessment by MRI^10^, ^25^, ^54^ and CT^54^ has been shown to be reproducible, a characteristic that should reflect in similar reproducibility for tortuosity. Indeed, descending aorta^37^ tortuosity assessment was found to be reproducible. Sequences such as black‐blood spin echo T1‐ or T2‐weighted ECG gated acquisitions allow for reproducible aortic wall thickness measure, with excellent intraobserver agreement^35^ and good‐to‐moderate^17^ and excellent^36^ interobserver agreement. Semiautomated measurement of aortic thickness on contrast‐enhanced CT angiography images showed excellent comparison with ex vivo specimens.^39^

#### Predictive Value

Aortic diameter has an independent prognostic value in the general population.^56^, ^57^, ^58^ While aortic length^54^ and tortuosity^59^ might hold predicting value for aortic adverse events, prospective studies testing their predictive value for cardiovascular diseases are missing. To the best of the authors' knowledge, studies assessing the predictive value of aortic wall thickness for cardiovascular diseases are lacking.

#### Limitations

One of the main limitations of geometry assessment in the vasculature is the poor standardization of the location of measurement, limiting the comparisons between studies, the availability of reference values and thus the generation of a solid background for further studies. Fully automatic techniques are likely to improve the workflow, improving standardization, reproducibility, and reduce the required time and expertise.^33^, ^41^

### Strain and Distensibility

Another main approach for the assessment of aortic stiffness relies on the quantification of the cycling deformation (strain) of the aortic wall because of pressure pulse or left‐ventricular shortening. Most of studies computed deformation measures from MRI (Figure 1), while only few from CT images^50^, ^60^, ^61^ Aortic distensibility, the most used potential biomarker in this category, exploits the existence of change in stress (pulse pressure) and quantifies the corresponding circumferential deformation. It is computed as the relative change in cross‐sectional area or diameter during the cardiac cycle divided by the local pulse pressure, and it is inversely related to aortic stiffness and PWV.^4^ By MRI, it is mostly computed in the mid‐ascending and descending thoracic aorta. Aortic stiffness index is another parameter built from the same theory, which differs from distensibility only for the logarithmic transformation of pressure, while circumferential strain, ie, the numerator of distensibility equation, is also often reported. Commonly, brachial pulse pressure instead of local pressure is adopted, introducing substantial errors.^62^ The error introduced by the incorrect estimation of local pulse pressure in the computation of distensibility and stiffness index remains to be established. In the last years there has been a growing interest in the assessment of longitudinal strain,^11^, ^50^, ^63^ especially in the proximal aorta,^11^, ^63^ where the left‐ventricle shortening forces aortic elongation. In both circumferential and longitudinal stain, a major concern is the absence of an accurate assessment of the intensity of the stress driving deformation.

#### Sequences

Distensibility, stiffness index and circumferential strain are mostly assessed on 2D SSFP or gradient echo “cine” (ie time‐resolved) MRI, with comparable results,^64^ and rarely by modulus images of PC MRI, which provide poorer delineation of aortic boundaries. Longitudinal deformation is mainly quantified in the proximal aorta by means of one or several cine MRI images. Advances in 3D time‐resolved SSFP sequences are likely to allow 3 and 4D assessment of arterial strain. In CT, time‐resolved images can be obtained by retrospective ECG gating.^60^, ^61^

#### Assessment

The assessment of arterial deformation is based either on manual segmentation or on feature tracking techniques, ie, image registration based on algorithms developed in the field of computer vision. For the computation of distensibility and stiffness index, changes in cross‐sectional area through the cardiac cycle are evaluated, with increased reproducibility with semiautomatic methods compared with manual ones.^64^ Conversely, several different methods to compute longitudinal deformation of the aorta have been reported. They all included cine MRI images of the proximal aorta, but they may include one view and compute local deformation^63^ or multiple perspectives and refer to anatomical landmark to quantify deformation.^11^ Conversely, longitudinal strain in the descending aorta by CT can be obtained assessing length at systole and diastole.^50^

#### Association With Age and Reference Values

Aortic distensibility is inversely related to age in children and adolescents^23^ and in adults,^6^, ^35^, ^50^, ^61^, ^64^, ^65^, ^66^, ^67^ with differences with respect to sex,^23^, ^65^, ^67^ especially in the proximal aorta.^66^ Lower values were reported in the descending compared with the ascending aorta.^64^ Age‐ and sex‐specific reference values for aortic distensibility in children and young adults have been reported in the ascending and descending aorta.^23^ Aortic circumferential strain also decreases with age.^50^, ^68^ Furthermore, longitudinal strain of the descending aorta decreases with age,^50^ but no data about other regions have been reported. Longitudinal strain of the proximal aorta is larger in women than in men.^63^

#### Accuracy and Reproducibility

Biomarkers of longitudinal and circumferential deformation have been shown to be inversely related to PWV.^6^, ^14^, ^63^, ^66^, ^67^ By MRI, intra‐ and interobserver reproducibility were acceptable^28^ or high,^63^, ^64^, ^66^ and scan‐rescan acceptable^28^ for distensibility in the thoracic aorta as well as for longitudinal deformation of the proximal aorta.^11^ Given the similarity in assessment, these results should translate to circumferential strain assessment as well. Despite resulting in similar values, inter‐observer reproducibility was higher when circumferential strain was assessed on cine compared with PC images.^64^ Minor influence of image spatial and temporal resolution on circumferential strain values was reported.^64^ Descending aorta distensibility by CT was reported reproducible.^60^, ^61^

#### Predictive Value

Ascending aorta distensibility predicts all‐cause mortality,^69^ hard cardiovascular events^69^ and nonfatal cardiac events^32^ in individuals without cardiovascular disease.

#### Limitations

The main practical limitations for distensibility and stiffness index relate to the poor correlation between acting forces (local pulse pressure) and the peripheral pulse pressure usually used for their assessment,^70^ while for strain measures it is the disregard of stress state. Moreover, despite substantial interaction shown between longitudinal and circumferential deformation in the proximal aorta,^63^ markers of circumferential stiffness are not often corrected for longitudinal deformation. Newer, 3D time‐resolved MRI or CT data are expected to allow automatic calculation of 3D deformation. The major drawback of time‐resolved CT is radiation exposure to patients.

### Calcium

Arterial calcification is a hallmark of arterial aging. Progressive deposits of calcium minerals in major arteries is associated with arterial stiffness, impaired hemodynamics, cardiovascular events, and mortality.^71^, ^72^ Arterial calcification is a systemic process that may be distributed to multiple vascular territories, with correlations between the extent of calcification in different vascular territories, including the aorta.^71^, ^72^, ^73^, ^74^, ^75^ CT is highly sensitive in detecting calcium and thus is the preferred imaging technique (Figure 2). Calcium can also be detected by MRI but low spatial resolution, prolonged acquisition time, and motion artifacts limit its use. Currently, coronary artery calcium (CAC) scoring by CT is recommended by clinical guidelines and widely used in clinical practice for cardiovascular risk assessment, while the assessment of calcification in other arteries is less established.^76^, ^77^

#### Imaging Protocol

Currently, CAC scoring is performed following a standardized protocol using multidetector CT scanners with electrocardiographic triggered data acquisition in diastole. A standardized tube voltage of 120 KVp, slice thickness of 3 mm and image matrix of 512×512 are selected, and the scan length is limited to the coverage of the heart, from the midlevel of the left pulmonary artery down to the diaphragm, during inspiratory breath hold. This protocol does not include in the field of view the aortic arch and the abdominal aorta, the aortic sites with the highest prevalence of calcium,^71^, ^74^, ^75^ being therefore, suboptimal for the quantification of aortic calcium. Further technological development including high‐pitch, selective attenuation, reconstruction algorithms, as well as motion correction have contributed to improve temporal resolution, reduce artifacts, and radiation doses.

#### Assessment

Semiautomatic CAC scoring is performed on axial slices of noncontrast or virtual noncontrast cardiac CT images. Calcification is identified as areas of attenuation >130 HU. Only contiguous pixels or voxels with area ≥1 mm^2^/mm^3^ or ≥3 adjacent pixels or voxels are counted as lesions to reduce the influence of image noise.^78^ CAC scoring is based on quantification of calcium load by different scoring algorithms:

1. *Agatston score*: The weighted sum of lesions area with a density >130 HU, multiplied by a density weighting factor that is derived from the maximal CT attenuation within a given calcified lesion (130–199 HU: factor 1, 200–299 HU: factor 2, 300–399 HU: factor 3 and >400 HU: factor 4).
2. *Calcium volume score*: Calculated by multiplying the number of voxels with calcification by the volume of each voxel to quantify calcium volume.
3. *Relative calcium mass score*: Calculated by multiplying the mean attenuation by the plaque volume in each image.

Conversely, a standard assessment of aortic calcification has not been established. The Agatston score is often used, but established strata developed for CAC are unlikely to be directly applicable to aortic calcium assessment.

#### Association With Age and Reference Values

The prevalence of coronary and aortic calcium increases with age, and varies with sex and ethnicity.^71^, ^72^, ^73^, ^79^ Age‐, sex‐ and race‐specific reference percentiles for the CAC Agatston score in different populations have been reported^76^, ^80^, ^81^ and suggested as a tool to calculate arterial age. Reference values from large populations are not available for other CAC scores nor for aortic calcium.

#### Accuracy and Reproducibility

The accuracy of CAC score in detecting coronary artery disease is high, in particular the negative predictive value, “zero calcium score”, is accurate. Inter‐ and intraobserver reproducibility is excellent for all CAC scores.^82^ The correlation between the different CAC scoring algorithms is excellent.^83^, ^84^ However, the CAC Agatston score is highly dependent on the scan protocol, scanner setting and attenuation threshold, while calcium volume score and the relative calcium mass score has proven to be more robust and reproducible methods.^78^

#### Predictive Value

CAC score is a marker of coronary atherosclerosis and associated with cardiovascular events and mortality. CAC score identifies subclinical coronary artery disease and improve cardiovascular risk assessment in asymptomatic subjects, in particular in low‐ to intermediate‐risk and in women.^72^, ^75^, ^85^, ^86^ Furthermore, asymptomatic subjects with a CAC score of zero have an excellent prognosis.^75^, ^87^ On the other hand, aortic calcification showed no or minimal independent prognostic value for hard cardiovascular events in several primary prevention cohorts when the aortic arch and abdominal aorta were excluded from the analysis.^72^, ^75^ Conversely, when the aortic arch or the abdominal aorta were analyzed, calcium was associated with all‐cause or cardiovascular mortality.^71^, ^75^, ^88^

#### Limitations

Overall, calcium evaluation is a reliable, noninvasive technique with a low risk of complications and high prognostic value. However, some characteristics and technical challenges may influence its clinical value. First, calcium assessment is associated with radiation exposure. However, the radiation dose, monitored by dose‐length‐product and effective radiation dose are low, typically <1.5 mSv for CAC, and larger for the larger field of view needed for aortic assessment. Second, CAC score, and likely aortic calcium metrics, is depending on machine settings, including tube voltage and timing of the triggered image acquisition in the cardiac cycle. Furthermore, beam hardening, partial volume effect and motion artifacts may interfere with calcium quantification. Third, the Agatston score assumes higher weighting at increasing calcium density, and fails to account for regional distribution, number and size of lesions, as well as calcification in other vascular territories.

## POSITRON EMISSION TOMOGRAPHY: A RELATED IMAGING TECHNIQUE

Combined with CT or MRI, positron emission tomography (PET) imaging permits the assessment of large arteries inflammation^89^ mainly by evaluating ^18^F‐fluorodeoxyglucose standardized uptake values (SUVs). SUV represents ^18^F‐fluorodeoxyglucose activity adjusted for dose, corrected for decay, and divided by body weight. To correct for background ^18^F‐fluorodeoxyglucose, whole artery SUV is either subtracted or divided (target to background ratio) by background SUV obtained from venous or remote arterial blood. ^18^F‐fluorodeoxyglucose SUV increases with age,^90^ predicts cardiovascular vents^89^, ^91^ and shows promise as a therapeutic target.^92^ Reference values have been reported.^93^ Nonetheless, the absence of standard in image acquisition and post‐processing pose challenges in obtaining comparable results among studies.

## OTHER POSSIBLE BIOMARKERS

MRI, CT, and PET allow for the assessment of other possible descriptors of aortic aging that were not included in the main list because they were either less established or because of the lack of prospective studies testing their prognostic value. Several other geometrical descriptors have been studied in the aorta. Aortic arch width, commonly defined as the distance between the ascending and descending aorta at the level of the pulmonary artery bifurcation, increases with age,^16^, ^94^ showed independent predictive values for cardiovascular events in the general population and its evaluation from cine MRI showed excellent reproducibility.^95^ Along with curvature, a geometrical description of local bending computed from local spatial derivatives of the centerline, they may become an important parameter to assess local arterial aging.^16^, ^94^ Beyond geometrical descriptors, comprehensive hemodynamic data such as those obtained by 4D flow MRI informed about the impact of aging on specific flow features,^21^ such as wall shear stress, which drives aortic remodeling and wall degeneration.^96^, ^97^ Moreover, a characterization of abdominal aorta stiffness able to capture aging can be achieved by MRI elastography.^18^ Furthermore, aortic calcification is prevalent in middle‐aged general population^79^ and its assessment demonstrated strong, independent predictive value for incident cardiovascular events.^73^, ^77^ Regarding PET, the use of ^18^F‐sodium fluoride as radiotracer can identify aortic microcalcifications, a possible precursor of calcific plaque.^98^

## PERSPECTIVE

Standardization, fundamental to build upon prior works, requires a compromise between continuous developments and improvements and the need for large scale, independent studies using the same setting and protocol. In this context, improvements in acquisition and reconstruction, and the automatization of certain tasks, such as segmentation^99^ and geometrical characterization,^100^ promise to reduce costs and improve image quality, resolution, and observer variability from the assessment process, which play a key role for further use of these imaging techniques in everyday clinical practice.

## CONCLUSIONS

MRI, CT, and PET provide detailed information on the status of deep arteries, both in terms of structural characteristics, such as dilation, calcification, elongation and tortuosity, and functional capacities, such as the pressure buffering function attributable to the distensibility of systemic arteries. Despite sharing common limitations, mainly cost and standardization, their use is growing and with it the knowledge of key pathophysiological processes involved in arterial aging and their clinical value as predictors of adverse cardiovascular events.

## Sources of Funding

This article is based upon work from COST Action CA18216 VascAgeNet, supported by COST (European Cooperation in Science and Technology, www.cost.eu). A. Guala has received funding from Spanish Ministry of Science, Innovation and Universities (IJC2018‐037349‐I) and from “la Caixa” Foundation (LCF/BQ/PR22/11920008). S. Piskin has received funding from the European Research Executive Agency, Marie‐Sklodowska Curie Actions‐Global Individual Fellowship (101038096), and from Istinye University, Scientific Research Projects project (2019B1). P. Wohlfahrt's work was supported by the Ministry of Health of the Czech Republic, grant no. NV 19‐09‐00125. J. Alastruey was supported by the British Heart Foundation under Grant PG/15/104/31913, the Wellcome Engineering and Physical Sciences Research Council Centre for Medical Engineering at King's College London under Grant WT 203148/Z/16/Z, and the UK Department of Health through the National Institute for Health Research Cardiovascular MedTech Co‐Operative at Guy's and St Thomas' National Health Service Foundation Trust under grant MIC‐2016‐019.

## Disclosures

Elisabetta Bianchini is cofounder of Quipu srl (Pisa, Italy), a spin‐off company of the Italian National Research Council and the University of Pisa. The remaining authors have no disclosures to report.
